# Novel Synthesis of 8-Deaza-5,6,7,8-tetrahydroaminopterin Analogues via an Aziridine Intermediate

**DOI:** 10.3390/molecules17055604

**Published:** 2012-05-10

**Authors:** Shouxin Zhou, Chao Tian, Chao Li, Ying Guo, Xiaowei Wang, Junyi Liu, Zhili Zhang

**Affiliations:** 1 Department of Chemical Biology, School of Pharmaceutical Sciences, Peking University, Beijing 100191, China; 2 State Key Laboratory of Natural and Biomimetic Drugs, Peking University, Beijing 100191, China

**Keywords:** tetrahydrofolate, aziridine, catalytic hydrogenation, solvent-free

## Abstract

An efficient method for the construction of the tetrahydrofolate skeleton is described. Starting from pterin analogues and aromatic amines, 8-deaza-5,6,7,8-tetrahydroaminopterin derivatives and the heterocyclic benzoyl isosteres were synthesized via a novel aziridine intermediate. Following this method, the byproducts of carbon-nitrogen bond hydrogenolysis in traditional synthetic strategy can be completely avoided.

## 1. Introduction

**Figure 1:**
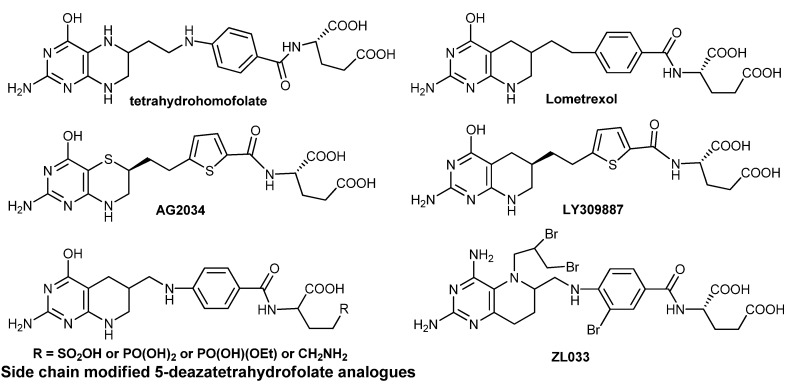
Tetrahydrofolate and tetrahydroaminopterin analogues.

Tetrahydrofolate plays a central role in one-carbon metabolism involving DNA biosynthesis, and the biologically active cofactor forms are substrates for at least 15 relevant enzymes [1,2,3,4]. Hence, tetrahydrofolate analogues are widely used as inhibitors of folate-dependent enzymes for chemotherapy. Early in 1960s, tetrahydrohomofolate (Figure 1) was found to be a potent inhibitor against TS (thymidine synthase) [5]. After then, more tetrahydrofolate analogues were developed as antitumor drugs, including lometrexol [6] as a specific inhibitor of GARFT (glycinamide ribonucleotide formyl transferase), AG2034 [7] and LY309887 [8] as next-generation GARFT inhibitors, side chain modified 5-deazatetrahydrofolate analogues [9] as potential dual inhibitors of FPGS (folylpolyglutamate synthetase) and GARFT as therapeutic agents, compound **1** and its analogues as MS (methionine synthase) and DHFR (dihydrofolate reductase) inhibitors [10].

**Scheme 1:**
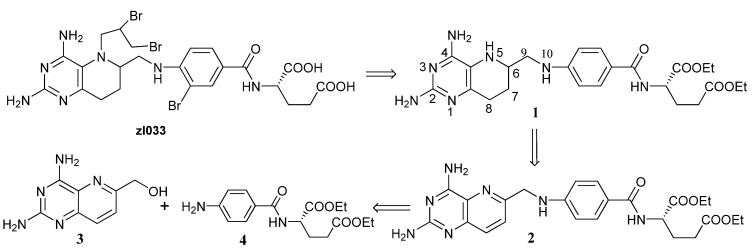
Reported route for compound **1**.

In our research on new antifolate inhibitors, we have identified *N*-[4-{(2-[2,4-diamino-5-(2,3-dibromopropane)-5,6,7,8-tetrahydropyrido(3,2-*d*) pyrimidin-6-yl]methyl)amino}-3-bromobenzoyl]-L-glutamic acid (ZL033) [10,11], which inhibits methionine synthase (IC_50_: 1.4 ± 0.4 µmol/L) and proliferation of HL-60 cells (IC_50_: 2.0 ± 1.0 µmol/L). Despite the remarkable biological importance of tetrahydrofolate analogues, not many synthetic approaches have been reported. The common synthesis strategy [12,13,14,15] is based on a critical step which is catalytic hydrogenation of folate derivatives, and was the strategy we used in synthesis of ZL033 and a series of novel 8-deazatetrahydrofolate derivatives as DHFR and MS inhibitors (Scheme 1). The reductive hydrogenation is a critical step in the entire synthetic scheme due to the benzylic hydrogenolysis of the C^9^-N^10^ bond as a side reaction. Careful adjustment of reaction solution acidity and protection of N^10^ with an electron-withdrawing substituent before reduction didn’t work well either. Against this background, we now report a practical and novel method for synthesizing 8-deazatetrahydrofolate **1** by ring-opening of an aziridine intermediate. Following this method, ZL033 and its heterocyclic benzoyl isosteres can be synthesized. And the modification in the side chain of 8-deazatetrahydrofolate can lead to more optional tetrahydrofolate analogues.

## 2. Results and Discussion

### 2.1. Retrosynthetic Analysis of ***1***

**Scheme 2:**
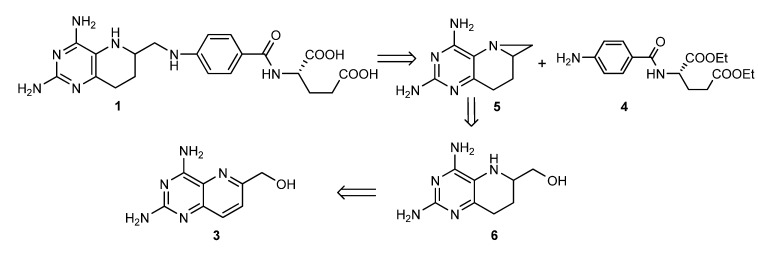
Synthetic strategy.

Our synthetic strategy is displayed in Scheme 2. Compound **1** is a key intermediate which can be converted into **ZL033** easily by the same method in Scheme 1, so we focused on the convenient synthesis of **1**. We envisaged that target compound **1** and its derivatives could be constructed by ring-opening of aziridine **5** with aromatic amines. The aziridine **5** would be synthesized via cyclization of compound **6**, which could be obtained by catalytic hydrogenation of compound **3**. Accordingly, we started our synthetic studies by seeking a general and practical method for preparing the reduced compound **6**.

### 2.2. Catalytic Hydrogenation of ***3***

**Scheme 3:**
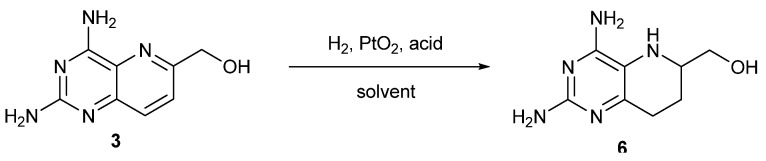
Catalytic hydrogenation of **3**.

Compound **3** was prepared in the same way as before [16,17], and was catalytically hydrogenated directly before linking with the side-chain amine to avoid C^9^-N^10^ hydrogenolysis (Scheme 3). As there is no fragile C^9^-N^10^ bond in **3**, stronger acidic conditions could be used compared to the similar hydrogenation in Scheme 1. An initial screening of the influence of different acidic conditions (Table 1) revealed that moderate acidity gave better results. The reduction reaction didn’t happen without acid added (Table 1, entry 1). Weak acids such as acetic acid gave product in 50~60% yield, even when excess acid was used as solvent (Table 1, entries 2~4), while the yield increased obviously with hydrochloric acid (Table 1, entries 5~7). Treatment of **3** with 8 equivalents of HCl for 24 h gave compound **6** in 93% yield, but with 16 equivalents of HCl, the yield was only 78%. The reason is that the hyroxymethyl group at 6-position of compound **3** could be reduced to 6-methyl group over PtO_2_ under strong acidic conditions. We actually got the 6-methyl isoster of compound **6** in 12% yield under the conditions of entry 7.

Variation of solvent also affected the yield strongly (Table 1, entries 6 and 8~9). Ethanol was a better solvent under these reaction conditions than methanol and acetone. The best conditions we found featured eight equivalents of hydrochloric acid in ethanol (Table 1, entry 6), resulting in a higher yield (93%) and the workup is easier than with the reduction reaction in Scheme 1. 

**Table 1:** Optimization of the catalytic reduction conditions ^a^.

Entry	Acid	Equiv.	Solvent	Time (h)	Yield (%) ^b^
1	-	-	Ethanol	120	-
2	AcOH	4	Ethanol	60	54
3	AcOH	8	Ethanol	60	47
4	AcOH	excess	AcOH	60	59
5	HCl ^c^	4	Ethanol	24	82
6	HCl ^c^	8	Ethanol	24	93
7	HCl ^c^	16	Ethanol	24	78
8	HCl ^c^	8	Methanol	8	21
9	HCl ^c^	8	Acetone	48	46

^a^ Conditions: Compound **3** (1 mmol), H_2_ (0.3 MPa), PtO_2_ (0.1 mmol), acid (4~16 mmol or as solvent), solvent (15 mL), H_2_O (0~8 mL, from HCl solution). ^b^ Yield of isolated product. ^c^ 2 mol/L aqueous HCl solution was used.

### 2.3. Optimized Synthesis of ***5***

Before trying this synthetic strategy, we attempted to directly synthesize **1** from **6** in several ways, but none of them succeeded. The hypothetical Mitsunobu reaction between **6** and diethyl *N*-(*p*-aminobenzoyl)-L-glutamate **4** wouldn’t proceed, and methods of transferring the hydroxymethyl group in **6** to aldehyde or halidemethyl group couldn’t improve this condensation reaction.

**Scheme 4:**
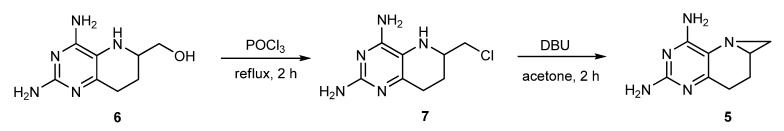
Two-step synthesis of compound **5**.

Inspired by the work of Singh [18], which described silica gel induced condensation of aziridines and aromatic amines, we designed aziridine **5** as the key intermediate [19,20,21]. Compound **6** was converted to the 6-chloromethyl derivative **7** in refluxing POCl_3_ (yield 40%), which was then treated with KOH, K_2_CO_3_ or 1,8-diazabicycloundec-7-ene (DBU) to give **5** in 86% yield after chromatographic purification, as shown in Scheme 4.

Compounds **7** and **5** were both identified by ^1^H-NMR, ^13^C-NMR and ESI-MS. The ^1^H-NMR (DMSO-*d*_6_) spectrum of **7** revealed a multiplet signal at δ 4.06~3.92 ppm assignable to CH_2_Cl, which obviously differed from the C*H*_2_OH group signal (δ 4.97~4.90 ppm) of compound **6**. The ^13^C-NMR (DMSO-*d*_6_) spectrum of **7** also displayed a different signal at δ 47.35 ppm assignable to CH_2_Cl with the CH_2_OH group signal at δ 51.79 ppm for **6**. The ESI-MS result of **7** gave the intuitive proof of a chloride atom by a pair of isotopic peaks (214.1 and 216.1, 3:1). The ^1^H-NMR (DMSO-*d*_6_) spectrum of **5** displayed two doublet signals at δ 2.14 ppm (d, *J* = 5.2 Hz, 1H) and 1.56 ppm (d, *J* = 4.0 Hz, 1H) which were readily assigned to the two hydrogen atoms attached at methylene position of the aziridine ring and the ^13^C-NMR (DMSO-*d*_6_) spectrum of **5** displayed a new signal at δ 33.33 ppm assignable to NCH_2_ in aziridine ring and a signal at δ 35.23 ppm assignable to NCH which gave both a relatively high-field shift compared to compounds **7** or **6**. High resolution MS provided further confirmation for the two products.

**Scheme 5:**
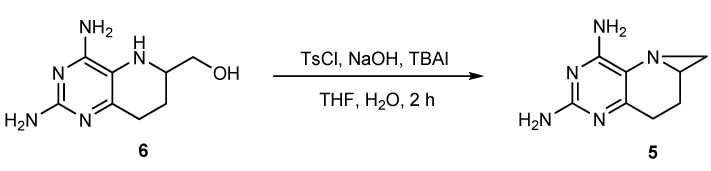
One-step synthesis of compound **5**.

The reaction above was somewhat low-yielding and its work-up procedure was complicated. After further investigation, we found an alternative method to obtain **5** in one step by treatment of **6** with *p*-toluenesulfonyl chloride, aqueous NaOH and tetrabutylammonium iodide in a two-phase system (Scheme 5). The yield increased to about 50%, and the work-up was simplified.

### 2.4. Synthesis of ***1***

**Scheme 6:**
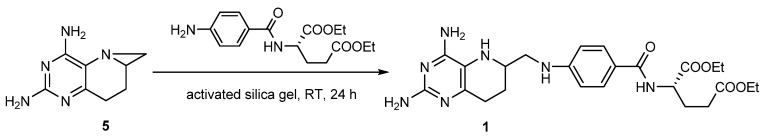
Synthesis of **1** under solvent-free conditions.

Compounds **5**, **4** and actived silica gel were mixed and shaken thoroughly under solvent-free conditions (Scheme 6). The desired product **1** was obtained in 90% yield, and the small amount of impurities could be removed easily by column chromatography. Compared with usual ring-opening reactions of aziridines using Lewis acid catalysis [22,23,24,25], the silica gel-induced method was convenient, green and cheap. Product **1** was verified by ^1^H-NMR and ESI-MS, and it was compared with the same compound prepared by Scheme 1 to prove the structural consistency.

### 2.5. Synthesis of ***ZL033***

**Scheme 7:**
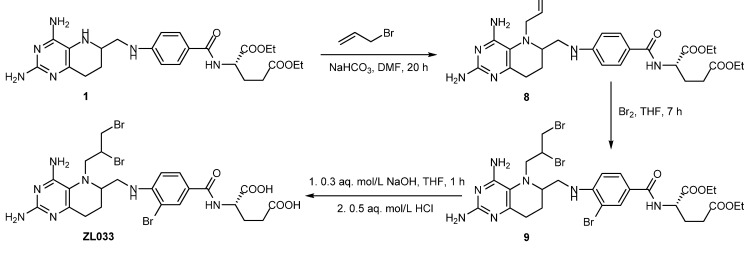
Synthesis of ZL033 from **1**.

Compound **1** can be converted to ZL033 in the same reaction sequence of allyl substitution, bromination and hydrolysis as before [11] (Scheme 7).

### 2.6. Synthesis of Other Tetrahydrofolate Analogues

**Scheme 8:**
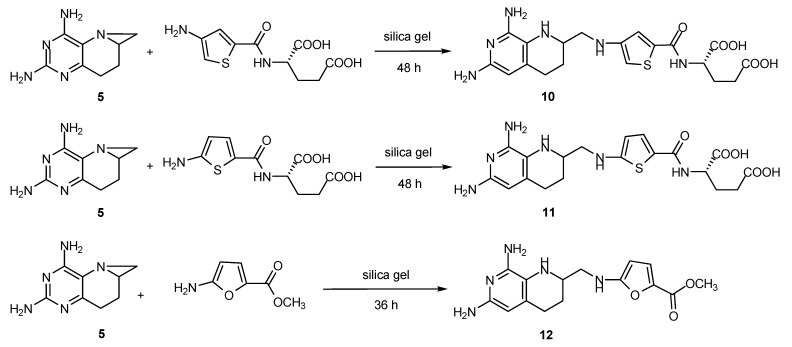
Synthesis of heterocyclic benzoyl analogues of **1**.

Aside from ZL033, many other tetrahydrofolate analogues can be synthesized from intermediate **1** by simply modifying the substituents on N^5^ or N^10^. Furthermore, the strategy above can be used for synthesis of various compounds, such as heterocyclic benzoyl analogues and modification in the side chain of tetrahydrofolate. Earlier research proved that substitution of N^10^-phenyl by aromatic heterocycles benefited inhibitory activity against several kinases [26,27,28,29]. The synthetic route of heterocyclic benzoyl analogues was similar with that of ZL033, including the ring-opening of aziridine by aromatic heterocyclic amines (Scheme 8). Compounds **10**, **11** and **12** were obtained in acceptable yields by the solid-phase reaction catalysed by silica gel.

Compounds **10**, **11** and **12** were transformed into analogues of ZL033 by substitution and hydrolysis reactions. This novel synthetic route is flexible to a certain extent. With various pteridines and aniline analogues, more tetrahydroaminopterin and tetrahydrofolate analogues were prepared in this novel method. The studies of these heterocyclic analogues of 8-deazatetrahydrofolate on the antifolate activity and the structure-activity relationship will be published in the future.

## 3. Experimental

### 3.1. General

All reactions were monitored by TLC analysis (pre-coated silica gel plates with fluorescent indicator UV254, 0.2 mm) and visualized with 254 nm UV light. The products were purified by column chromatography on silica gel (silica gel 230–400 mesh or silica gel H, produced by Qingdao Ocean-Chem Inc., Qingdao, China), or preparative thin layer chromatography on silica gel (silica gel GF_254_, 0.5 mm, also produced by Qingdao Ocean-Chem Inc., Qingdao, China). ^1^H-NMR spectra were measured on Bruker avance III 400 spectrometer (400 MHz, Bruker Co., Fällanden, Switzerland) or JEOL JNM-AL300 spectrometer (300 MHz, Joel, Japan). Data were reported as follows: Chemical shifts in ppm from tetramethylsilane as an internal standard in CDCl_3_ or DMSO-*d*_6_, multiplicity (s = singlet, d = doublet, t = triplet, q = quartet, m = multiplet, br = broad), coupling constants (Hz), integration. ^13^C-NMR spectra were recorded on a Bruker Avance III 400 (100 MHz) or JEOL JNM-AL300 spectrometer (75 MHz). Mass spectra were measured with a Quattro micro 2000 (ESI-TOF) instrument (Bruker Co., Bremen, Germany). HRMS was performed on a Bruker APEX IV FT-MS (7.0T, Waters, New Castle, DE, USA). Melting points were measured with a SGW X4 microscopic melting point apparatus. Chemicals were purchased from Aldrich, Acros, AlfaAsar, Strem, OuheChem or Beijing Chemical Works and were used without further purification, unless otherwise noted. Hydrogen gas needed in reduction reaction was supplied by a GCD-300A hydrogen generator, manufactured by the Beijing BCHP Analytical Technology Institute.

*(2*,*4-Diamino-5*,*6*,*7*,*8-tetrahydropyrido[3,2-d]pyrimidin-6-yl)methanol* (**6**): To a suspension of 2,4-diamino-6-hydroxymethylpyrido*[3,2-d]*pyrimidine (**3**, 2.01 g, 10.5 mmol) in ethanol (150 mL) was added aqueous HCl (40 mL, 2 mol/L, 80 mmol) and platinum dioxide monohydrate (250 mg, 1.0 mmol). The mixture was then hydrogenated in a 0.3 MPa hydrogen generator at room temperature for 24 h. After evaporation of solvents and acid, anhydrous ethanol (300 mL) was added to the residue and stirred for 1 h. The resulting precipitate was filtered, washed with acetone (100 mL) and dried under vacuum to afford 1.90 g (92.9% yield) of **6** as a white solid; m.p. 241~243 °C. ^1^H-NMR (300 MHz, DMSO-*d*_6_): δ = 7.90 (br.s, 2H, 4-NH_2_), 6.93 (br.s, 2H, 2-NH_2_), 4.97~4.90 (br.s × 2, 2H, C*H*_2_OH), 3.47~3.41 (m, 1H, 6-CH), 3.16 (m, 1H, 8-C*H*_a_H_b_), 2.68~2.59 (m, 1H, 8-CH_a_*H*_b_), 1.93~1.88 (m, 1H, 7-C*H*_a_H_b_), 1.53 (m, 1H, 7-CH_a_*H*_b_); ^13^C-NMR (75 MHz, DMSO-*d*_6_): δ = 157.47 (C-8a), 150.52 (C-4), 129.18 (C-2), 116.39 (C-4a), 63.92 (CH_2_OH), 52.74 (C-6), 22.41 (C-7, C-8); MS (ESI): *m/z* 196.1 (M+H^+^, 100%); HRMS: calcd for C_8_H_14_N_5_O [M+H]^+^ 196.11984, found 196.11963.

*2*,*4-Diamino-6-choloromethyl-5*,*6*,*7*,*8-tetrahydropyrido[3,2-d]pyrimidine* (**7**): A stirred solution of compound **6** (502 mg, 5.1 mmol) in POCl_3_ (10 mL) was refluxed at 110 °C for 2 h. The resulting solution was evaporated to a minimized volume of about 1 mL under reduced pressure. Then it was diluted with 50 mL dichloromethane and evaporated to a minimized volume again. This procedure was repeated twice with diluting solvent changing successively to ethanol and methanol. Acetone (50 mL) was added to the concentrated sticky liquid, and the resulting white precipitate was filtered, washed with acetone (20 mL) and dried under vacuum to afford 221 mg (40.2% yield) of **7** as an off-white solid; m.p. 228~232 °C. ^1^H-NMR (400 MHz, DMSO-*d*_6_): δ = 4.06~3.92 (m, 2H, CH_2_Cl), 3.70~3.68 (m, 1H, 6-CH), 2.64~2.44 (m, 2H, 8-CH_2_), 1.98~1.95 (m, 1H, 7-C*H*_a_H_b_), 1.77~1.70 (m, 1H, 7-CH_a_*H*_b_); ^13^C-NMR (100 MHz, DMSO-*d*_6_): δ = 51.79 (6-CH), 47.35 (CH_2_Cl), 23.19 (7-CH_2_), 22.04 (8-CH_2_); MS (ESI): *m/z* 214.1 (M+H^+^, isotopic peak of **^37^Cl**, 100%), 216.1 (M+H^+^, isotopic peak of **^35^Cl**, 33.6%); HRMS: calcd for C_8_H_13_ClN_5_ [M+H]^+^ 214.08595, found 214.08617.

2,4-Diamino-5,6-methylene-5,6,7,8-tetrahydropyrido[3,2-d]pyrimidine (**5**)

Method A: To a suspension of compound **7** (296 mg, 1.4 mmol) in acetonitrile (25 mL) was added 1,8-diazabicyclo[5.4.0]undec-7-ene (DBU, 0.60 mL, 4.1 mmol). The reaction was stirred at room temperature for 1 h, then concentrated and isolated by preparative TLC with a chloromethane and methanol mixture (9:1) as eluent. Compound **5** (210 mg) was obtained as a faint yellow solid, the yield was 85.7%, while the two-step overall yield from compound **6** was 34.5%.

Method B: To a suspension of compound **6** (300 mg, 1.5 mmol) in anhydrous THF (10 mL) was added 50% aq. sodium hydroxide solution (4.0 mL, 76.3 mmol) and tetrabutylammonium iodide (101 mg, 0.3 mmol). *p*-Toluenesulfonyl chloride (291 mg, 1.5 mmol) was added after stirring for 10 min. The reaction mixture was stirred at room temperature for 1 h, and diluted with ethyl acetate (100 mL) and stirred thoroughly. The resulting solution was then filtered through kieselguhr and washed with dichloromethane (50 mL). The filtrate and washes were combined, concentrated and subjected to preparative TLC with a dichloromethane and methanol mixture (9:1) as eluent. Compound **5** (137 mg, 50.3%) was obtained as a faint yellow solid; m.p. 223~226 °C. ^1^H-NMR (400 MHz, DMSO-*d*_6_): δ = 6.12 (s, 2H, 4-NH_2_), 5.52 (s, 2H, 2-NH_2_), 2.48~2.47 (m, 1H, 6-CH), 2.34~2.28 (m, 1H, 8-C*H*_a_H_b_), 2.27~2.19 (m, 1H, 8-CH_a_*H*_b_), 2.14~2.13 (d, *J =* 5.2 Hz, 1H, NC*H*_a_H_b_), 2.09~2.06 (m, 1H, 7-C*H*_a_H_b_), 1.96~1.91 (m, 1H, 7-CH_a_*H*_b_), 1.56~1.55 (d, *J =* 4.0 Hz, 1H, NCH_a_*H*_b_); ^13^C-NMR (100 MHz, DMSO-*d*_6_): δ = 160.50 (2-C), 159.09 (4-C), 154.62 (8a-C), 117.48 (4a-C), 35.23 (6-CH), 33.33 (N-CH_2_), 25.26 (8-CH_2_), 21.05 (7-CH_2_); MS (ESI): *m/z* 178.1 (M+H^+^, 100%); HRMS (ESI): calcd for C_8_H_12_N_5_ [M+H]^+^ 178.10872, found 178.10813.

*Diethyl N-{4-[(2*,*4-diamino-5*,*6*,*7*,*8-tetrahydropyrido[3,2-d]pyrimidin-6-yl)methylamino]benzoyl}-L-glutamate* (**1**): Silica gel was activated at 120 °C under vacuum for 6 h before use. Compound **5** (139 mg, 0.8 mmol) and diethyl 4-aminobenzoyl-L-glutamate (310 mg, 1.0 mmol) were mixed uniformly with activated silica gel (1.02 g). The mixture was stirred vigorously for 24 h, and stopped when TLC showed full transformation of **5**. The reactant was purified by column chromatography (silica gel). Elution with CHCl _3_-CH_3_OH (12:1) gave pure **1** (356 mg, 90.8%) as a white solid; m.p. 143 °C (decomp). ^1^H-NMR (400 MHz, DMSO-*d*_6_): δ = 8.27~8.25 (d, *J =* 7.3 Hz, 1H, CONH), 7.69~7.67 (d, *J =* 8.7 Hz, 2H, 2,6-H on benzene ring), 7.29 (s, 2H, 4-NH_2_), 6.67~6.64 (d, *J =* 8.7 Hz, 2H, 3,5-H on benzene ring), 6.55~6.53 (d, *J =* 8.5 Hz, 1H, 5-NH), 6.50~6.47 (t, *J =* 5.7 Hz, 1H, CH_2_N*H*), 4.40~4.35 (m, 1H, CONHC*H*), 4.11~4.02 (m, 4H, C*H*_2_CH_3_ × 2), 3.68~3.65 (m, 1H, 6-CH), 3.21~3.17 (m, 2H, C*H*_2_NH), 2.43~2.39 (m, 4H, 8-CH_2_, CHCH_2_C*H*_2_), 2.12~1.97 (m, 4H, 7-CH_2_, CHC*H*_2_CH_2_), 1.18~1.16 (m, 6H, CH_3_ × 2); MS (ESI): *m/z* 499.3 (M+H^+^, 100%); HRMS (ESI): calcd for C_24_H_34_N_7_O_5_ [M+H]^+^ 500.26159, found 500.26051.

*Diethyl N-{4-[(2*,*4-diamino-5*,*6*,*7*,*8-tetrahydropyrido[3,2-d]pyrimidin-6-yl)methylamino]thiophene-2-formyl}-L-glutamate* (**10**): The heterocyclic benzoyl analogue **10** was prepared in the similar way as the synthesis of **1**. Compound **5** (144 mg, 0.8 mmol), diethyl *N*-(4-aminothiophene-2-formyl)-L-glutamate (600 mg, 1.8 mmol) and activated silica gel (1.6 g) was stirred together for 48 h to complete the reaction. Product was purified by column chromatography (silica gel). Elution with CHCl_3_-CH_3_OH (15:1) gave pure **10** (186 mg, 45.3%) as a faint yellow liquid. ^1^H-NMR (400 MHz, CDCl_3_): δ = 7.16 (s, 1H, 2-H on thiophene ring), 6.10 (s, 1H, 4-H on thiophene ring), 5.21 (br.s, 1H, 5-NH), 4.72 (br.s, 1H, CH_2_N*H*), 4.58 (br.s, 1H, CONHC*H*), 4.18 (m, 2H, CHCOOC*H*_2_), 4.10~4.09 (m, 3H, CH_2_COOC*H*_2_, 6-CH), 3.31 (m, 1H, C*H*_a_H_b_NH), 3.14 (m, 1H, CH_a_*H*_b_NH), 2.64~2.35 (m, 4H, 8-CH_2_, CHCH_2_C*H*_2_), 2.27~2.10 (m, 4H, 7-CH_2_, CHC*H*_2_CH_2_), 1.24~1.22 (m, 6H, CH_3_ × 2); HRMS (ESI): calcd for C_22_H_32_N_7_O_5_S [M+H]^+^ 506.21801, found 506.21906. 

*Diethyl N-{5-[(2*,*4-diamino-5*,*6*,*7*,*8-tetrahydropyrido[3,2-d]pyrimidin-6-yl)methylamino]thiophene-2-formyl}-L-glutamate* (**11**): The heterocyclic benzoyl analogue **11** was prepared in the same way as the synthesis of **10**. Compound **5** (96 mg, 0.5 mmol), *N*-(5-aminothiophene-2-formyl)-L-glutamate (403 mg, 1.2 mmol) and activated silica gel (1.05 g) was stirred together for 48 h to complete the reaction. Product was purified by column chromatography (silica gel). Elution with CHCl_3_-CH_3_OH (15:1) gave pure **11** (120 mg, 43.8%) as a faint yellow liquid. ^1^H-NMR (400 MHz, CDCl_3_): δ = 7.26 (s, 1H, 2-H on thiophene ring, coincided with CHCl_3_ peak), 6.42 (s, 1H, 3-H on thiophene ring), 5.30 (s, 1H, 5-NH, coincided with residual CH_2_Cl_2_ peak), 4.63 (m, 2H, CH_2_N*H*, CONHC*H*), 4.18~4.17 (m, 2H, CHCOOC*H*_2_), 4.09~4.08 (m, 2H, CH_2_COOC*H*_2_), 3.60 (m, 1H, 6-CH), 3.41~3.35 (m, 2H, C*H*_2_NH), 2.65~2.64 (m, 1H, 8-C*H*_a_H_b_), 2.43 (m, 2H, CHCH_2_C*H*_2_), 2.27~2.26 (m, 1H, 8-CH_a_*H*_b_), 2.22~2.17 (m, 1H, CHC*H*_a_H_b_CH_2_), 2.08 (m, 2H, CHC*H*_a_H_b_CH_2_, 7-C*H*_a_H_b_), 1.91 (m, 1H, 7-CH_a_*H*_b_), 1.22~1.19 (m, 6H, CH_3_ × 2); HRMS (ESI): calcd for C_22_H_32_N_7_O_5_S [M+H]^+^ 506.21801, found 506.21947.

*Methyl 5-[(2*,*4-diamino-5*,*6*,*7*,*8-tetrahydropyrido[3,2-d]pyrimidin-6-yl)methylamino]furan-2-carboxylate* (**12**): The heterocyclic benzoyl analogue **11** was prepared in a similar way as in the synthesis of **1**. Compound **5** (90 mg, 0.5 mmol), methyl 5-aminofuran-2-formate (181 mg, 1.3 mmol) and activated silica gel (501 mg) was stirred together for 36 h to complete the reaction. Product was purified by column chromatography (silica gel). Elution with CHCl_3_-CH_3_OH (15:1) gave pure **12** (104.5 mg, 40.7%) as a faint yellow liquid. HRMS (ESI): calcd for C_14_H_19_N_6_O_3_ [M+H]^+^ 319.15186, found 319.15244. 

## 4. Conclusions

In conclusion, we have developed a novel synthetic route providing the biologically important compound **1** in good yields via an aziridine intermediate, thus avoiding the benzylic hydrogenolysis of the carbon-nitrogen bond of earlier methods. It also represents a convenient preparation of tetrahydroaminopterin or tetrahydrofolate analogues.
